# The Modification of Activated Carbon for the Performance Enhancement of a Natural-Rubber-Based Triboelectric Nanogenerator

**DOI:** 10.3390/polym15234562

**Published:** 2023-11-28

**Authors:** Pongsakorn Mekbuntoon, Sirima Kongpet, Walailak Kaeochana, Pawonpart Luechar, Prasit Thongbai, Artit Chingsungnoen, Kodchaporn Chinnarat, Suninad Kaewnisai, Viyada Harnchana

**Affiliations:** 1 Department of Physics, Faculty of Science, Khon Kaen University, Khon Kaen 40002, Thailand; pongsakorn_m@kkumail.com (P.M.); sirima.ko@kkumail.com (S.K.); walailakkaeochana@kkumail.com (W.K.); pawonpart_l@kkumail.com (P.L.); pthongbai@kku.ac.th (P.T.); 2 Institute of Nanomaterials Research and Innovation for Energy (IN-RIE), Khon Kaen University, Khon Kaen 40002, Thailand; 3 Department of Physics, Faculty of Science, Mahasarakham University, Maha Sarakham 44150, Thailand; artit.c@msu.ac.th (A.C.); chinnarat.sphrt@gmail.com (K.C.); suni.suninad@gmail.com (S.K.)

**Keywords:** triboelectric nanogenerator, activated carbon, acid treatment, N_2_ plasma, natural rubber

## Abstract

Increasing energy demands and growing environmental concerns regarding the consumption of fossil fuels are important motivations for the development of clean and sustainable energy sources. A triboelectric nanogenerator (TENG) is a promising energy technology that harnesses mechanical energy from the ambient environment by converting it into electrical energy. In this work, the enhancement of the energy conversion performance of a natural rubber (NR)-based TENG has been proposed by using modified activated carbon (AC). The effect of surface modification techniques, including acid treatments and plasma treatment for AC material on TENG performance, are investigated. The TENG fabricated from the NR incorporated with the modified AC using N_2_ plasma showed superior electrical output performance, which was attributed to the modification by N_2_ plasma introducing changes in the surface chemistry of AC, leading to the improved dielectric property of the NR-AC composite, which contributes to the enhanced triboelectric charge density. The highest power density of 2.65 mW/m^2^ was obtained from the NR-AC (N_2_ plasma-treated) TENG. This research provides a key insight into the modification of AC for the development of TENG with high energy conversion performance that could be useful for other future applications such as PM2.5 removal or CO_2_ capture.

## 1. Introduction

Due to a predominant reliance on limited and non-renewable energy sources, significant issues have arisen concerning energy shortages and environmental pollution. Consequently, clean and sustainable energy has received continuous attention and development. The triboelectric nanogenerator (TENG) is an innovative technology capable of harvesting mechanical energy and converting it into electrical energy, based on the principles of contact electrification and electrostatic induction [1]. TENG has been demonstrated to be highly efficient, environmentally friendly, low-cost, easy to manufacture, and suitable for large-scale applications. Its versatility extends to various applications, such as sensors [2], portable micro/nano power sources [3], raindrop energy harvesting [4], and air filtration systems [5].

Natural-based materials, such as leaves, wood, silk, and paper, have been developed for TENG applications. Most of them have shown promising energy production performance and potential applications. However, some of them experience difficulties in the modulation of their intrinsic property to enhance the power output of TENG due to processing constraints.

Natural rubber (NR) is a natural polymeric material with a chemical structure of cis-1,4-polyisoprene. NR latex is typically extracted from *Hevea brasiliensis* trees, which are grown in tropical areas such as Southeast Asia, India, and Africa. NR possesses many outstanding characteristics, including high elasticity, low thermal conductivity, good electrical insulation, and biodegradability [6]. In this regard, NR has been developed for diverse applications including examination gloves, car wheels, packaging, and shoes. In addition, NR film is made from NR latex, which is feasible for manufacturing processes and modifications such as the incorporation of filler materials to engineer the properties of the NR film.

NR-TENG has been developed and is gaining more attention. Various modification approaches have been proposed for improving the performance of NR-TENG by intensifying the triboelectric charge density on friction layers. These modifications include adding metal/metal oxide nanoparticles such as silver (Ag) [7,8], TiO_2_ nanoparticles [9], hybrid Ag–cellulose [10], and activated carbon (AC) [10,11]. These filler materials were found to enhance the charge capacitance of the NR triboelectric layer through dielectric constant modulation via the interfacial polarization of conductive nanoparticles in a dielectric NR polymer [12]. Other conductive nanostructured materials have been reported to effectively enhance the power output of polymer-based TENGs such as Au [13] and carbon nanotubes [14]; however, they are costly, which could be a major constraint for large-scale production and commercialization. Among these filler materials, AC is a carbon material with a highly porous structure, giving it incredibly high specific surface area, making it an excellent adsorbent for a wide range of substances [15], leading to a broad spectrum of applications, including in air and water filtration/purification [16], CO_2_ adsorption [17], environmental remediation [18], supercapacitors [19], and medical [20] and pharmaceutical [21] areas. In this regard, AC is a promising filler for NR since a large surface area is a key benefit required for enhancing the triboelectric charge density [22,23]. Moreover, AC is inexpensive and offers numerous ways to further modify surface structure and chemistry [24].

Many different methods have been proposed for modifying the surface of activated carbon, such as acid treatment, base treatment, impregnation treatment, ozone treatment, surfactant treatment, plasma treatment, and microwave treatment [24]. For acid treatments, HCl [25], H_2_SO_4_ [26], and HNO_3_ [27] have been reported. KOH [28], NaOH [29], and NH_3_ [30,31] are used as base treatments. In addition, other substances such as ZnCl_2_ [32], NaNH_2_ [33], and NH_4_Cl [34] are also used for the treatment. Furthermore, plasma treatments such as O_2_ [35], CO_2_ [36], and N_2_ [37,38] have also been reported.

Apart from surface morphology modifications, surface functional groups are important for increasing the surface charge density of TENG by increasing the ability to gain or lose electrons [39]. It has been confirmed by many studies that acid/base treatments are able to change surface chemistries when various chemical functional groups are introduced to AC materials [40,41]. For example, single-bond oxygen functional groups were obtained from HCl modification, carbonyl carboxyl and nitrate groups from HNO_3_, sulfur-containing groups from H_2_SO_4_ treatment, and a hydroxyl group from NaOH treatment [40]. In addition to the wet chemical process with acid/base treatments, the surface functional groups of AC materials can be modified by N_2_ plasma treatment, which introduces not only a micropore structure but also nitrogen functional groups to AC material [38].

In this work, the effects of a surface treatment method on the surface morphology, specific surface area, and surface chemistry of an AC material are investigated. According to the results from a previous study, it was shown that the acid treatments gave better improvement in terms of specific surface area compared to the basic treatment [42]. In this study, we use acid treatments with three different types of acid (HCl, H_2_SO_4_, and HNO_3_), and N_2_ plasma treatment to modify the surface of AC. These modified AC particles are subsequently used as fillers in NR material, aiming to enhance the energy production of TENG. The correlations of the specific surface area of AC, dielectric properties, and TENG performance of the NR-AC are studied. Furthermore, the applications of the fabricated NR-AC TENG as a power source for small electronic devices are demonstrated. This work has proposed an effective approach to enhance the energy conversion performance of the natural-based TENGs, addressing critical challenges in the development of large-scale renewable energy sources that are environmentally friendly and sustainable.

## 2. Materials and Methods

### 2.1. AC Modified with Acid Treatment

A commercial AC powder was purchased from SIGMA-ALDRICH, St. Louis, Missouri, USA. To achieve finer particles, the AC power underwent a 24-h ball milling process before either subsequent acid or plasma treatment. This initial AC material is referred to as AC (ball mill). A total of 37% hydrochloric acid (HCl, RCI Labscan, Bangkok, Thailand), 98% sulphuric acid (H_2_SO_4_, ANaPURE, Brightchem Sdn. Bhd., Selangor, Malaysia), and 65% nitric acid (HNO_3_, ANaPURE, Brightchem Sdn. Bhd., Selangor, Malaysia) were employed in the modification of AC powder. A total of 5 g of AC powder was mixed with a 50 ml acid solution and magnetically stirred for 6 h. Subsequently, the acid-treated products were washed with deionized (DI) water until they reached a pH of 5. The resulting materials were then dried at 150 °C overnight. The specimens were labelled as AC (HCl), AC (H_2_SO_4_), and AC (HNO_3_), corresponding to the types of acid used.

### 2.2. AC Modified with N_2_ Plasma Treatment

Nitrogen plasma with a pressure of 1.68 Torr was used to treat AC powders. AC powders were placed in a stainless steel lunchbox and positioned on the powered electrode. Plasmas were generated using an asymmetric bipolar pulse power supply (DC Pinnacle^®^ Plus+, Advanced Energy, Shanghai, China) set at 500 W power, a frequency of 50 kHz, and a duty cycle of 10%. The treatment was sustained for 60 s.

### 2.3. Preparation of NR–AC Composite Film

The modified AC powder from each treatment, at a concentration of 0.4% (*w*/*v*), was added to 20 mL of NR latex obtained from the Thai Rubber Latex Group Public Co., Ltd. (Chonburi, Thailand), with a dry rubber content of 61%. The selection of 0.4% AC addition aligned with the optimum fraction determined in our previous work [11]. The mixtures underwent magnetic stirring for 20 min to obtain homogeneous suspensions. A 2.0 mL portion of the mixture was cast onto a 4 × 4 cm^2^ ITO substrate to produce a film thickness of approximately 0.7 mm. Three samples were prepared for each experimental condition. The specimens were then allowed to air-dry at room temperature for 1 day before being cured at 60 °C for 6 h. Subsequently, the samples were ready for the TENG performance test.

### 2.4. Material Characterizations

The morphologies and crystal structures of the modified AC powders and NR-AC composite films were examined using scanning electron microscopy (SEM) (Helios Nanolab, FEI, Waltham, MA, USA) and X-ray diffraction (XRD) (PANalytical EMPYREAN, Malvern, UK), respectively. The specific surface area was analyzed by the Brunauer–Emmett–Teller (BET) analysis, and the pore size distribution was analyzed using the N_2_-DFT method. Micropore and mesopore volumes were derived from the t-plot (TP) and Barrett–Joyner–Halenda (BJH) methods, respectively. The chemical functionalities of the specimens were characterized via Fourier-transform infrared spectroscopy (FTIR TENSOR27, Bangkok, Thailand).

### 2.5. TENG Output Measurement

The TENG performance of NR-AC composites was evaluated using a single electrode mode with a contact-separation configuration. A PTFE sheet with a lateral dimension of 4 × 4 cm^2^ and a thickness of 5 mm was used as a contact material. The separation distance between the triboelectric layer was 8 cm. The generated electrical outputs voltage and current were acquired using an oscilloscope (Tektronix DPO2002B, Tektronix China Ltd., Shanghai, China) with an input impedance of 10 MΩ and a digital ammeter (Keithley DMM6500, Tektronix China Ltd., Shanghai, China), respectively. Data were collected under an applied impact force of 3 N at a frequency of 5 Hz. The delivered power density of TENGs was determined by connecting a series of external load resistances ranging from 0.5 to 10 MΩ.

## 3. Results

**Figure 1:**
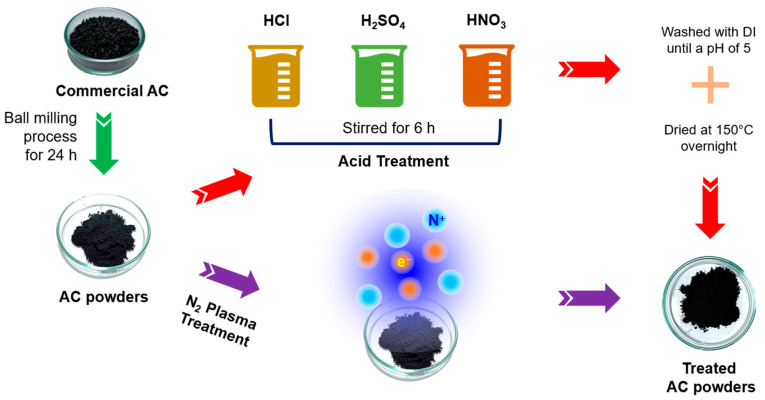
Schematic diagram of the experiment using two different techniques: acid treatment and N_2_ plasma treatment to modify the AC powders.

**Figure 2:**
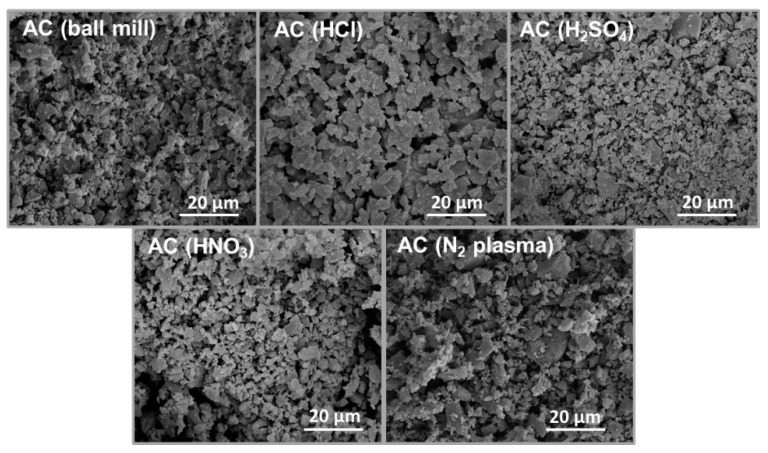
SEM images of AC (ball mill), AC (HCl), AC (H_2_SO_4_), AC (HNO_3_), and AC (N_2_ plasma) powders.

A schematic diagram in Figure 1 shows a summary of the two different treatment methods employed to modify AC powders. SEM images in Figure 2 reveal the microstructures of the modified AC particles: AC (ball mill), AC (HCl), AC (H_2_SO_4_), AC (HNO_3_), and AC (N_2_ plasma). The particle sizes of the acid-treated and N_2_-plasma-treated AC specimens are relatively similar, with an average particle size comparable to that of the ball-milled AC (AC (ball mill)), except for HCl acid-treated AC, where the particles aggregated, resulting in a larger particle size. 

The specific surface area, total pore volume, and average pore size were analyzed and displayed in Table 1. It was shown that the modification of AC by acid treatment and plasma treatment did not induce significant changes in surface area and pore structure, except for the AC(HNO_3_), which exhibited a slightly lower specific surface area and total pore volume. This discrepancy was attributed to the larger pore diameter compared to other samples. However, these alternations in specific surfaces were not found to be significant, consistent with findings from previous studies [40].

**Figure 3:**
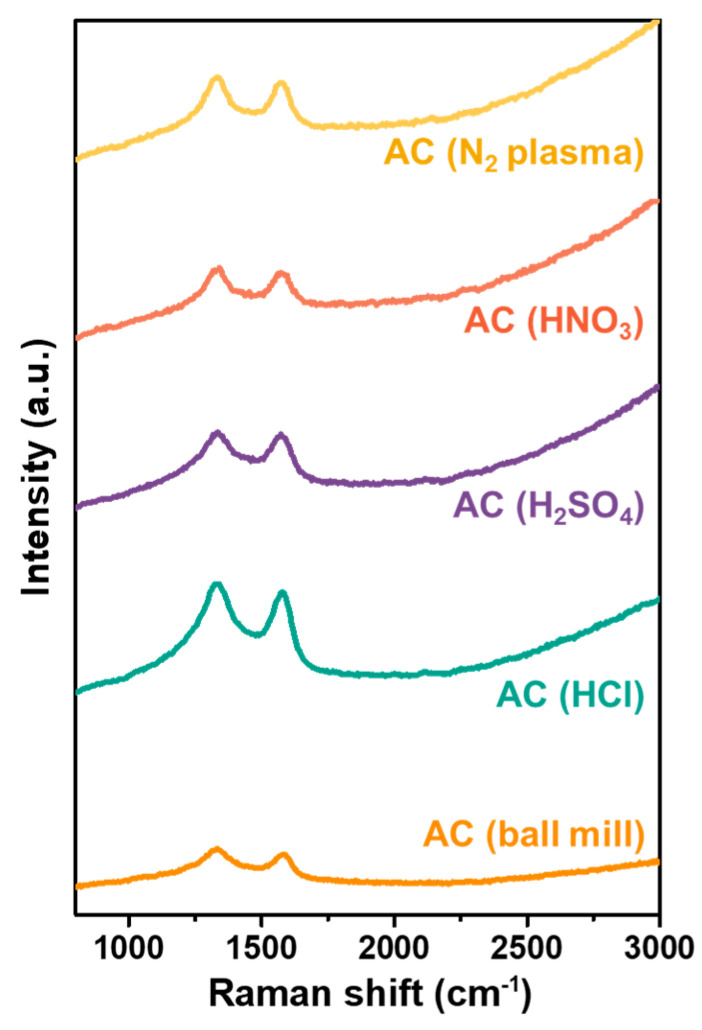
Raman spectra of the unmodified AC powders (ball mill), and the modified AC including the acid-treated and N_2_ plasma-treated AC.

The Raman spectra of all AC samples, including AC (ball mill) and the modified AC samples, are displayed in Figure 3. Two characteristic peaks of carbon are evident in all specimens at 1335 cm^−1^ and 1575 cm^−1^, assigned to the D and G peaks, respectively. These Raman spectra resemble those of activated carbon reported in other previous work [43].

**Figure 4:**
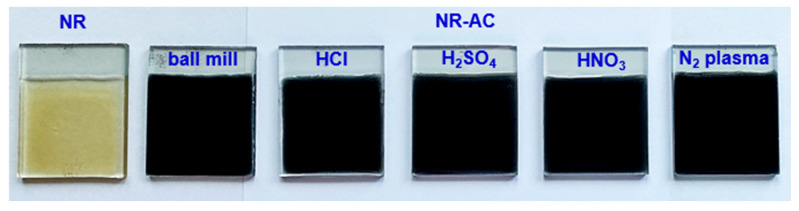
Digital photographs of pristine NR films (without AC) and NR–AC composite films on ITO substrates including NR-AC (ball mill), NR-AC (HCl), NR-AC (H_2_SO_4_), NR-AC (HNO_3_), and NR-AC (N_2_ plasma).

The NR and NR-AC composite films coated onto ITO-conductive glass substrates used for fabrication TENG are revealed in Figure 4. The NR composite, incorporating modified AC particles including AC (ball mill), AC (HCl), AC (H_2_SO_4_), AC (HNO_3_), and AC (N_2_ plasma), maintained a uniform black color characteristic of AC, whereas pristine NR film appeared transparent with light yellow color. All NR-AC composite films exhibit a homogeneous dispersion of AC particles, and the aggregation of AC particles into large clusters was not visible on the film surfaces.

**Figure 5:**
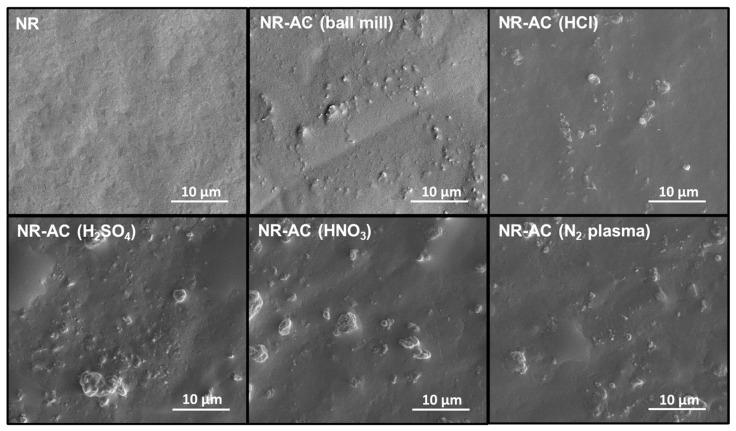
Surface of pristine NR film, NR-AC (ball mill), NR-AC (HCl), NR-AC (H_2_SO_4_), NR-AC (HNO_3_), and NR-AC (N_2_ plasma) composite films.

The surface morphologies of NR and all NR-AC composite films are presented in Figure 5. AC particles are clearly visible on the surfaces of all NR-AC composite films without significant differences, whereas the surface of NR appears flat with no observed particles. The sizes of AC particles in the composites are consistent with those observed in the SEM images of the AC particles in Figure 2.

**Figure 6:**
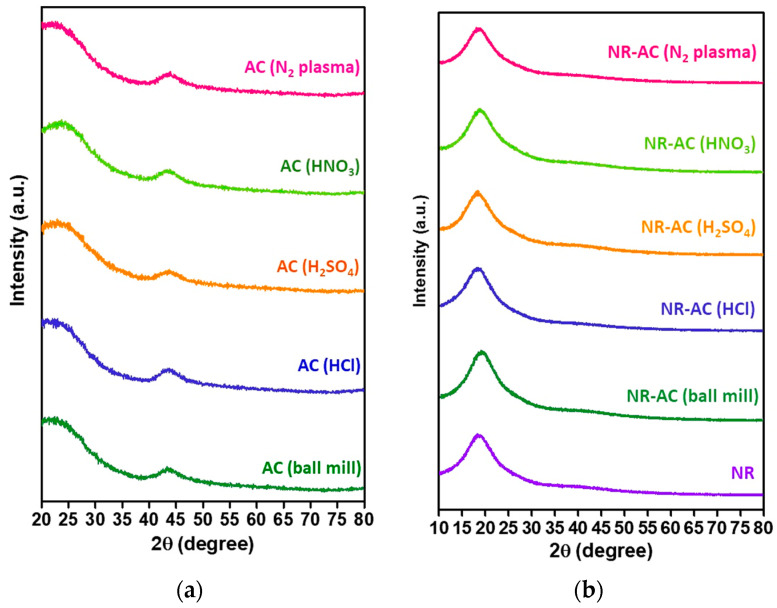
(**a**) XRD patterns of the unmodified AC (AC (ball mill) and modified AC particles including AC (HCl), AC (H_2_SO_4_), AC (HNO_3_), and AC (N_2_ plasma). (**b**) XRD of all the NR-AC composite films.

The microstructures of the AC particles and NR-AC composite films were also probed using XRD analysis, as shown in Figure 6. The XRD patterns of all the modified AC particles in Figure 6a exhibited a consistent broad diffraction peak at 2θ~43°, indicating an amorphous carbon structure and the modification did not alter the crystal structure of AC. In the case of NR-AC composite films, broad peaks at 18° were observed in all XRD patterns (Figure 6b), corresponding to diffraction peaks from amorphous NR, consistent with findings from previous studies [44,45]. The diffraction peak from AC at ~43° was not observed in the XRD patterns of the composite films due to the small fraction of AC present in the composite (0.4%).

**Figure 7:**
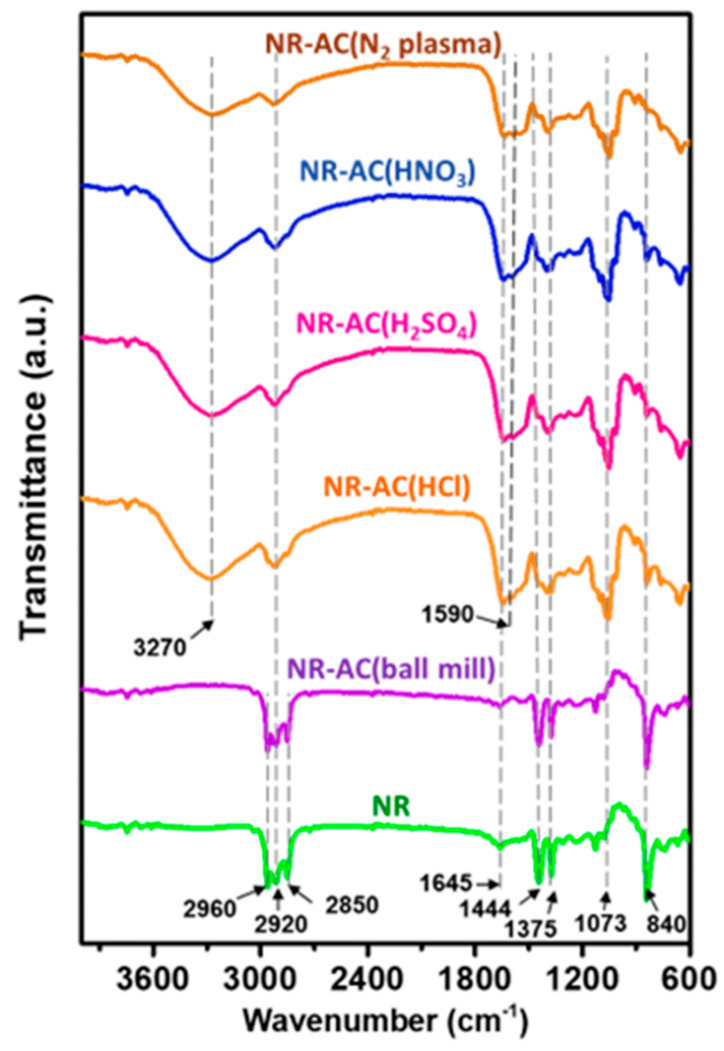
FTIR spectra of pristine NR film and NR–AC composite films.

The chemical structures of the fabricated composite films were inspected using FTIR analysis. FTIR spectra of pristine NR and NR–AC are presented in Figure 7. The FTIR spectra of pristine NR and NR-AC (ball mill) are similar but differ from those of NR-modified AC composites, which are almost identical. All NR composites show absorption peaks at 840 and 1645 cm^−1^, assigned to out-of-plane bending vibrations of C–H and C=C stretching of a cis-1,4-polyisoprene molecule of NR, respectively. The peaks at 1375 and 1444 cm^−1^ are associated with O-H bending vibration from water and C-H bending vibration from the methyl group, respectively. These peaks are more pronounced in the FTIR spectra of NR and NR-AC (ball-mill) composites than those in the NR-modified AC composites, so the multiple peaks at 2850–2920 and 2960 cm^−1^ correspond to the asymmetric–symmetric stretching vibration of CH_2_ and C–H in NR molecule, respectively. For all NR composites with modified AC, broad peaks at 3270 cm^−1^ and 1590 cm^−1^ are detected, associated with the stretching vibration of O–H from carboxylic acid and C=C from cyclic alkene in AC particles. These peaks are absent in the pristine NR and NR-AC (ball-mill) specimens, suggesting that the modification processes caused a change in the chemical structures of AC particles.

**Figure 8:**
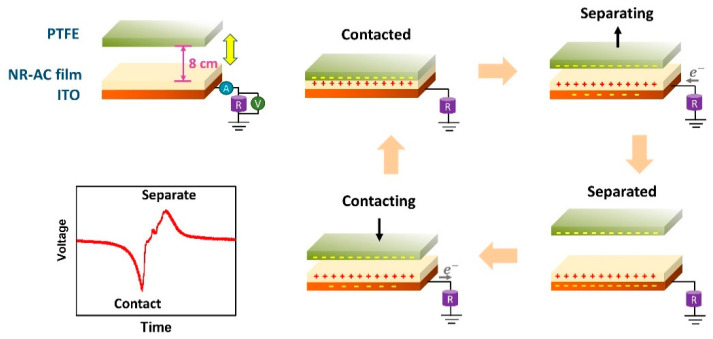
Schematic diagram presenting the working mechanism of NR composite TENG in a single-electrode mode with the inset of the generated electrical signal.

The electrical generation performance of the fabricated TENGs was examined using NR-AC films on ITO glasses (Figure 4) as the bottom positive tribo-electrode for TENG and a PTFE sheet as the contact tribo-negative materials. Performance testing was carried out under a consistent impact force of 3 N with a frequency of 5 N. The electricity generation during a contact-separation movement is illustrated in Figure 8. Upon initial contact between the surfaces of the NR-AC film and PTFE, electrons are transferred from the NR-AC composite to PFTE due to the electrification effect, resulting in the formation of positive surface charges on NR-AC and negative surface charges on PTFE surfaces. The subsequent separation of the two materials with opposite surface charges leads to the buildup of electrical potential, inducing free electrons to flow from the ground to the ITO glass, counterbalancing this potential and resulting in the generation of a positive current signal. When the two surfaces are brought back in contact, the potential decreases and disappears, causing electrons to return to the ground, thereby generating a negative current, as illustrated in an inset.

**Figure 9:**
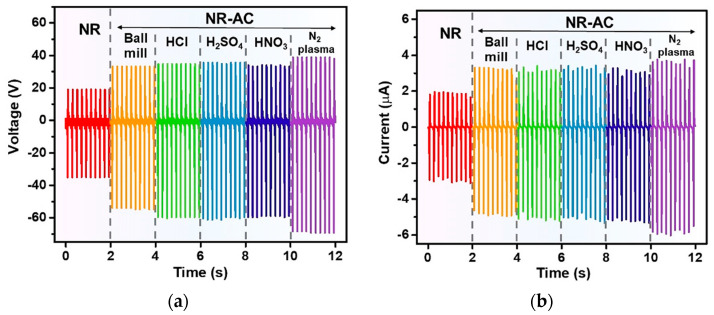
(**a**) Output voltage and (**b**) current of the fabricated TENGs from NR and NR–AC composite films tested under the 3N impact force at a working frequency of 5 Hz.

The measured electrical outputs of the fabricated TENG are presented in Figure 9a,b, representing output voltage and current, respectively. It was seen that the incorporation of untreated (AC ball mill), acid-treated, as well as plasma-treated AC into NR substantially increased the electrical outputs of the TENGs. All acid-treated-AC TENGs exhibited a similar trend in electrical outputs, slightly lower than those of the N_2_ plasma-treated one. The NR–AC (N_2_ plasma) TENG showed the highest peak-to-peak voltage (*V_pp_*) of 108 V and peak-to-peak current (*I_pp_*) of 9.8 µA, whereas the NR-AC(H_2_SO_4_), NR-AC(HCl) and NR-AC(HNO_3_) TENGs had *V_pp_* of 97, 95, and 94 V, and *I_pp_* of 8.7, 8.6, and 8.5 µA, respectively. These TENGs exhibited superior energy conversion performance compared to the NR-AC (ball mill) and pristine NR TENGs, as summarized in Table 2 for *V_pp_* and *I_pp_* of all the fabricated TENGs.

**Figure 10:**
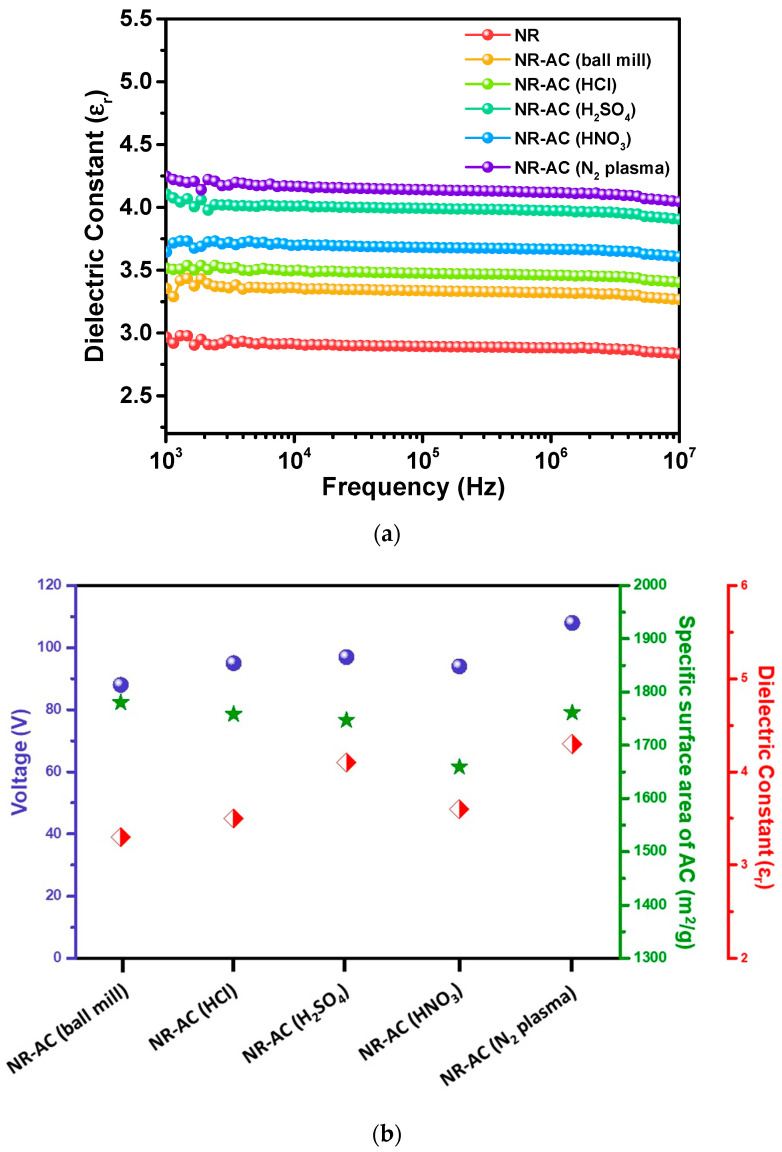
(**a**) Dielectric constant of pristine NR and NR-AC composite films at various frequencies measured at room temperature. (**b**) Plot of TENG electrical voltage output, specific surface area, and dielectric constant of the NR-AC composites.

To elucidate the effect of AC surface modification on TENG performance, the dielectric constants of the specimens were examined. The dielectric constant indicates charge storing ability of the sample, which can be used to represent the changes in chemical structure resulting from surface modification processes. The plot of dielectric constant versus frequencies at room temperature is presented in Figure 10a. The results reveal that the NR-AC (N_2_ plasma) composite had the highest dielectric constant, followed by NR-AC(H_2_SO_4_), NR-AC(HNO_3_), NR-AC(HCl), NR-AC (ball mill), and pristine NR, respectively. The dielectric constants exhibited a relatively similar trend to that of the electrical outputs, as presented by the plot correlating TENG output performance, specific surface area, and dielectric constants of all specimens in Figure 10b. Despite the nearly unchanged specific surface areas, the modified AC, including NR-AC (N_2_ plasma) and all the NR-acid-treated AC TENGs, showed higher electrical outputs than the untreated AC. This suggests that surface modifications did not significantly alter the surface morphologies but led to changes in surface chemistries, which in turn increased the triboelectric charge density, as evidenced by the enhanced electrical output.

While FTIR and elemental analysis did not provide substantial information regarding changes in the chemical functional groups and elemental composition resulting from surface treatments, alteration in dielectric constant implies an enhanced capacity to retain triboelectric charges. This enhancement could be attributed to changes in the chemical and electronic properties of the surfaces. This aligns with the findings from previous studies on surface modification involving nitrogen and oxygen functional groups, known to promote the electron-donating ability of triboelectric materials [13,46].

**Figure 11:**
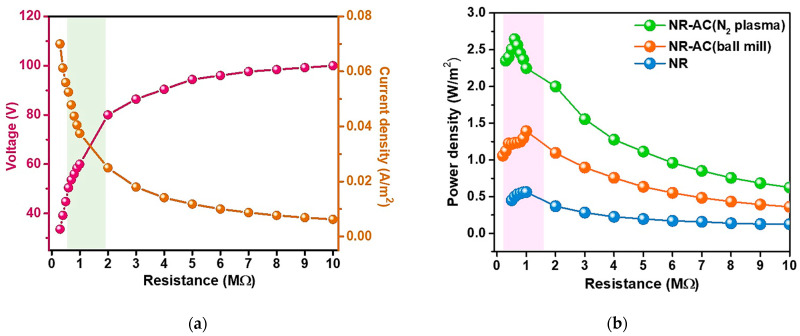
(**a**) Dependence of output voltage and current on the connected load resistances of the NR–AC (N_2_ plasma) TENG and (**b**) power densities of NR–AC (N_2_ plasma) TENG compared to those of NR and NR–AC (ball mill) TENGs.

The performance of electrical power generation of the NR-AC (N_2_ plasma) TENGs was further evaluated in terms of electrical power density. The electrical voltage and current outputs of the TENG were measured across a range of load resistances from 0.5 to 10 MΩ. The plots of the measured output voltage versus current at various load resistances are displayed in Figure 11a. Power density was subsequently calculated using the formula *P_d_* = *V* × *J*, where *J* represents current density (*J* = *I/area*). The plot of power density at various load resistances of the NR-AC (N_2_ plasma) TENGs, compared to those of NR and NR-AC (ball mill) TENGs, is presented in Figure 11b, and their maximum power densities with the corresponding matched load resistances are presented in Table 3. The NR–AC (N_2_ plasma) TENG exhibited the highest power density of 2.65 W/m^2^, surpassing those of NR-AC (ball mill) and NR TENGs, which were 1.39 and 0.56 W/m^2^, respectively. The performance of the NR-AC TENG in this work showed superior output performance compared to many reported natural-based TENGs, as shown in Table 4 [47,48,49,50,51,52,53,54,55,56,57,58].

It was also observed that the matched load of the NR-AC (N_2_ plasma) TENG was lower than those of NR and NR-AC (ball mill) TENGs, suggesting that the NR–AC (N_2_ plasma) composite had lower internal resistance. This was attributed to the fact that N_2_ plasma generates electric charges, including free radicals and electrons during the treatment. Typically, three possible events occur on the material surface when treated with N2 plasma; (i) a change in surface roughness with a hierarchical nanostructure, (ii) improved hydrophilic properties, and (iii) the introduction of nitrogen-containing functional groups [59]. In this work, the surface roughness (surface area) did not undergo dramatic changes (Table 1), and nitrogen-containing functional groups were not detected via EDS and FTIR. However, it was possible that the AC powders treated with a high power of bipolar pulse plasma resulted in bond breaking, producing free radicals and electrons [60]. In this regard, N_2_ plasma is considered a powerful technique for modifying the surface chemistry of AC, leading to the superior electrical output of TENG.

**Table 4:** Summary of power output performances of TENGs fabricated from natural-based materials compared to the NR-AC TENG from this study [61].

Natural-Material-Based TENGs	Power Density	Applications	References
Natural rubber-activated carbon/PTFE	2.65 W/m^2^	Energy harvesting	This work
silk fibroin/rice paper	21.6 mW/m^2^	Power implantation device	[47]
Chitosan/Kapton	2.1 µW/m^2^	Energy harvesting	[48]
Silk/Si-rubber	16.6 μW/cm^2^	Energy Harvesting	[49]
fish gelatin/PTFE-coated PDMS	45.8 μW/cm^2^	Energy Harvesting	[50]
cyclo-phenylalanine peptide/PTFE	73.7 mW/m^2^	Energy harvesting	[51]
Cellulose/FEP	14 μW/cm^2^	Power electronics device	[52]
Ag-doped Cellulose/FEP	7.68 µW/cm^2^	Air filter and Antibacterial patch	[53]
BaTiO_3_-doped bacteria cellulose/PDMS	4.8 W/m^2^	Human motion energy harvesting	[54]
Cellulose/Nitrocellulose	16.1 W/m^2^	Paper piano	[55]
Cellulose/PTFE	18.4 W/m^2^	Velocity and Force Sensor	[56]
Leaf/PVDF	1.1 mW/cm^2^	Energy harvesting	[57]
Rice paper/PVC	37.64 μW/cm^2^	Power electronics device	[58]

**Figure 12:**
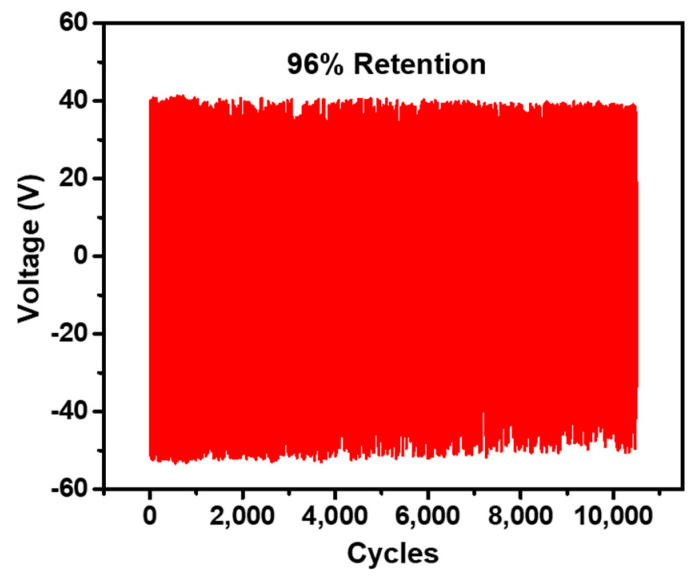
Electrical output voltage of the NR-AC (N_2_ plasma) TENG measured over 10,000 cycles.

The working stability of the NR-AC (N_2_ plasma TENG) TENG was assessed by measuring the output retention under the impact force of 3 N at 5 Hz frequency, as presented in Figure 12. The NR-AC composite TENG demonstrated consistent output retention over 10,000 cycles or approximately 30 min. This indicates that AC powder possesses a notable ability to retain charges and maintain film deformation, contributing to the overall stability of the NR composite TENG.

**Figure 13:**
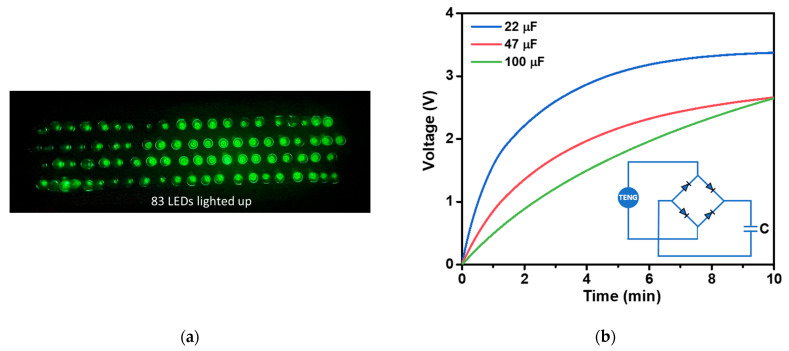
(**a**) Demonstration of the NR-AC TENG a power source for illuminating 83 green LEDs. (**b**) Voltage profiles of 22, 47, and 100 µF capacitors charged by the fabricated NR–AC TENG with a bridge rectifier.

The applications of the fabricated TENG were demonstrated as a power source, capable of directly powering small electronics such as lighting up LED or charging commercial capacitors, as presented in Figure 13a,b, respectively. A total of 83 commercial green LEDs were successfully illuminated by the instantaneously generated electrical power from the TENG (Figure 13a). The TENG was able to charge commercial capacitors with capacitances of 22, 47, and 100 µF through a bride rectifier, taking 55, 143, and 240 s to reach 1.5 V, respectively (Figure 13b). Furthermore, with the high surface area and good adsorption properties of AC materials, the modified AC embedded in NR film could be further developed as a triboelectric filter membrane for the removal of PM2.5 [62], CO_2,_ and NO_2_ gases [17]. This presents a significant challenge for future research on TENG.

## 4. Conclusions

The AC modified via acid treatment and N_2_ plasma treatment were found to enhance the performance of the NR TENG. Despite causing no significant change in specific surface area, all modifications resulted in an increase in dielectric constant, which correlated with TENG performance. The NR composite with AC particles modified by N_2_ plasma exhibited the highest electrical output. This achievement was attributed to the modified chemical structure caused by the high power of plasma radiation, introducing free radicals and electrons creating dipole formation in the NR composite film. This modification also reduced the internal resistance of the NR-AC composite film compared to both pristine NR and the NR-AC (unmodified). The highest power density of 2.65 W/m^2^ was achieved from the NR-AC (N_2_ plasma) TENG, surpassing that of the pristine NR TENG by 4.5 times.

## Figures and Tables

**Table 1:** Specific surface area, total pore volume, and average pore size of the modified AC particles: AC (ball mill), AC (HCl), AC (H_2_SO_4_), AC (HNO_3_), and AC (N_2_ plasma).

Specimens	*S_BET_* (m^2^/g)	*V_total_* (cm^3^/g)	Average Pore Diameter (nm)
AC (ball mill)	1780	0.874	1.96
AC (HCl)	1758	0.859	1.95
AC (H_2_SO_4_)	1747	0.855	1.96
AC (HNO_3_)	1659	0.813	2.35
AC (N2 plasma)	1762	0.865	1.96

**Table 2:** Electrical output voltage (*V_pp_*) and current (*I_pp_*) of NR and NR–AC TENGs.

Specimens	*V_pp_* (V)	*I_pp_* (µA)	Dielectric Constant (*ɛ_r_*)
Pure NR	54	5.0	2.9
NR–AC (ball mill)	88	8.2	3.4
NR–AC (HCl)	95	8.6	3.5
NR–AC (H_2_SO_4_)	97	8.7	4.1
NR–AC (HNO_3_)	94	8.5	3.6
NR–AC (N_2_ plasma)	108	9.8	4.3

**Table 3:** Maximum power density of NR, NR–AC (ball mill), and NR–AC (N_2_ plasma) TENGs with their corresponding matched loads.

TENGs	Power Density (W/m^2^)	Matched Load (MΩ)
Pure NR	0.56	1.0
NR–AC (ball mill)	1.39	1.0
NR–AC (N_2_ plasma)	2.65	0.6

## Data Availability

Data are contained within the article.
